# Children with oligoarticular juvenile idiopathic arthritis have skewed synovial monocyte polarization pattern with functional impairment—a distinct inflammatory pattern for oligoarticular juvenile arthritis

**DOI:** 10.1186/s13075-020-02279-9

**Published:** 2020-08-12

**Authors:** Tobias Schmidt, Elisabet Berthold, Sabine Arve-Butler, Birgitta Gullstrand, Anki Mossberg, Fredrik Kahn, Anders A. Bengtsson, Bengt Månsson, Robin Kahn

**Affiliations:** 1 Department of Pediatrics, Clinical Sciences Lund, Lund University, BMC B13, Klinikgatan 26, 22185 Lund, Sweden; 2 Wallenberg Center for Molecular Medicine, Lund University, BMC B13, Klinikgatan 26, 22185 Lund, Sweden; 3 Department of Rheumatology, Clinical Sciences Lund, Lund University, Lund, Sweden; 4 Department of Infection Medicine, Clinical Sciences Lund, Lund University, Lund, Sweden

**Keywords:** Juvenile idiopathic arthritis, Monocytes, Polarization, Inflammation, Synovitis

## Abstract

**Background:**

Juvenile idiopathic arthritis (JIA) is an umbrella term of inflammatory joint diseases in children. Oligoarthritis is the most common form in the Western world, representing roughly 60% of all patients. Monocytes and macrophages play an important role in adult arthritides, but their role in oligoarticular JIA is less studied. Polarization highly influences monocytes’ and macrophages’ effector functions, broadly separated into pro-inflammatory M1 or anti-inflammatory M2 phenotypes. Here, we set out to investigate the polarization pattern and functional aspects of synovial monocytes in oligoarticular juvenile idiopathic arthritis (JIA).

**Methods:**

Paired synovial fluid, blood samples (*n* = 13), and synovial biopsies (*n* = 3) were collected from patients with untreated oligoarticular JIA. Monocytes were analyzed for polarization markers by flow cytometry and qPCR. Effector function was analyzed by a phagocytosis assay. Polarization of healthy monocytes was investigated by stimulation with synovial fluid in vitro. Monocyte/macrophage distribution, polarization, and mRNA expression were investigated in biopsies by immunohistochemistry, immunofluorescence, and in situ hybridization.

**Results:**

Children with oligoarticular JIA have polarized synovial fluid monocytes of a specific M1(IFNγ)/M2(IL-4)-like pattern. This was evidenced by increased surface expression of CD40 (*p* < 0.001), CD86 (*p* < 0.001), and CD206 (*p* < 0.001), but not CD163, as compared to paired circulating monocytes. Additionally, polarization was extensively explored at the mRNA level and synovial fluid monocytes differentially expressed classical markers of M1(IFNγ)/M2(IL-4) polarization compared to circulating monocytes. Synovial fluid monocytes were functionally affected, as assessed by reduced capacity to phagocytose (*p* < 0.01). Synovial fluid induced M2 markers (CD16 and CD206), but not M1 (CD40) or CD86 in healthy monocytes and did not induce cytokine production. Single and co-expression of surface CD40 and CD206, as well as mRNA expression of IL-10 and TNF, was observed in monocytes/macrophages in synovial biopsies.

**Conclusion:**

Children with untreated oligoarticular JIA have similar and distinct synovial fluid monocyte polarization pattern of mixed pro- and anti-inflammatory features. This pattern was not exclusively a result of the synovial fluid milieu as monocytes/macrophages in the synovial membrane show similar patterns. Our study highlights a distinct polarization pattern in oligoarticular JIA, which could be utilized for future treatment strategies.

## Introduction

Juvenile idiopathic arthritis (JIA) is a heterogenous inflammatory disease mainly affecting the joints. JIA is an umbrella description of arthritides persisting longer than 6 weeks with unknown etiology in patients below 16 years of age [1]. The international league of associations for rheumatology (ILAR) recognizes seven categories of JIA [2]. Oligoarticular JIA is the most common subgroup, affecting 50–80% of JIA patients and involves fewer (≤ 4) and mainly larger joints at presentation [3, 4]. The majority of these patients have auto-antibodies against nuclear components (ANA), but lack rheumatoid factor (RF) [3]. These children are typically of preschool age when a disease develops [3]. Distinct adult counterparts exist for all JIA subgroups; however, the most common form of JIA, ANA-positive oligoarthritis with a high risk of uveitis, is uncommon.

The pathogenesis of JIA is mostly unknown. Synovium is exposed to the infiltration of leukocytes, hyperplasia, and long-lasting inflammation. Amongst the infiltrating cells are monocytes, which are plastic and heterogenous cells categorized based on CD14 and CD16 surface expression into “classical” (CD14^+^CD16^−^), “intermediate” (CD14^+^CD16^+^), and “alternative” (CD14^−^CD16^+^) subtypes [5]. “Classical” monocytes are the most prevalent, representing approximately 80–90% of the circulating monocytes under healthy conditions. In arthritis, monocytes are also found within synovial fluid and tissue where they demonstrate clear differences to their circulating counterparts, e.g., by increased CD16 expression [6].

Monocytes and macrophages are highly influenced by the surrounding environment, which may polarize—or activate—these cells, resulting in specialized effector functions. This polarization process traditionally separates them in vitro into pro-inflammatory M1 or anti-inflammatory M2 phenotypes [7, 8]. In vitro, monocyte- or macrophage phenotypes are induced by various cytokine cocktails. The M1 phenotype is usually induced with IFNγ and/or LPS, whilst the M2 phenotype is achieved using IL-4 or IL-10. Here, we refer to these phenotypes as M1(IFNγ), M2(IL-4), and M2(IL-10), respectively, as endpoints of polarization [7, 9]. However, it is important to remember that this classification is, amongst others, a mere in vitro simplification, and represents only endpoints in a continuum of polarization. M1(IFNγ) monocytes have an increased surface expression of CD40 and CD80 and produce pro-inflammatory cytokines, such as IL-1β and TNF [7, 9]. These cells have also been shown to have decreased Fc-mediated phagocytosis [10]. In contrast, M2(IL-4) monocytes express high levels of CD206, TGM2, and PPARγ [11]. They produce anti-inflammatory cytokines, including TGF-β and IL-10. Finally, M2(IL-10) are highly phagocytic and efferocytic, expressing CD163, as well as CD16 and HO-1 [12].

Monocytes simultaneously showing M1- and M2-like properties are identified in vivo, seemingly highly context-dependent. Polarization is believed to influence disease by affecting effector functions [13]. For example, IL-10 positive M2(IL-10)-like macrophages have been found in the lining layer of the synovium in rheumatoid arthritis (RA) and, more prominently, in spondylarthritis (SpA), whilst the sub-lining layers consisted more of a heterogenous M1/M2-like pattern [14]. Stimulation of monocyte-derived macrophages with synovial fluid from patients with SpA induced a higher expression of M2 markers CD163 and CD200R1 than synovial fluid from patients with RA [15]. Circulating and synovial intermediate CD14^+^CD16^+^ monocytes are expanded in enthesitis-related JIA, where synovial monocytes display a M2(IL-10)-like pattern [16, 17]. Thus, M2(IL-10) polarization patterns have been associated with arthritides. Nevertheless, the precise role of M1/M2 monocytes and macrophages in arthritis remains to be identified, and to our knowledge, there have been no studies thoroughly investigating the polarization of monocytes in oligoarticular JIA.

Here, we aimed to map the polarization state of synovial monocytes and macrophages in oligoarticular JIA. We speculated that monocytes voyaging from the circulation to the synovium polarizes in both the inflamed tissue and synovial fluid and contribute to the pathogenesis through cytokine production, cell-cell interactions, and reduced phagocytosis.

## Methods

### Patient data, materials, and sample collection

Patients with oligoarticular JIA without any antirheumatic medications with active disease undergoing therapeutic joint aspiration at the Department of Pediatrics, section for Pediatric Rheumatology, between 2016 and 2019, were included in the study. As patients routinely are prescribed non-steroid anti-inflammatory drugs (NSAIDs) by the primary care, these drugs were not considered as treatment of JIA. In total, 16 patients were included in this study. Two patients were later found to have another diagnosis or subclassification and were thus excluded from the analysis. Blood (*n* = 13) and synovial fluid (*n* = 13) samples were collected. Synovial fluid was aspirated from knee joints in all patients. When possible, synovial biopsies (*n* = 3) were taken by an ultrasound guided technique as previously described [18]. Details on biopsy handling are found in supplementary material. Details on sample handling are found in supplementary material. Patient data and performed analyses on specific patients can be found in Table 1. This study was approved by the Regional Ethical Review Board for southern Sweden (LU2016/128 and LU2017/473). Informed consent was obtained from the patients and the patient’s guardians.

**Table 1:** Clinical data and sample information of the included patients

	Sex M/F	Disease duration (months)	Age^**+**^ (years)	Treatment	Uveitis	ANA	SF/plasma	Biopsy	Cytokine assay SF/plasma	Phagocytosis
**1**	F	0	6	NSAID	Yes	Pos	Y/N	Yes	Y/N	Yes
**2**	F	3	11	No	Yes	Pos	Y/Y	No	Y/Y	Yes
**3**	F	2	3.5	No	No	Pos	Y/Y	Yes	Y/Y	Yes
**4**	M	1	12	NSAID	No	Neg	Y/Y	No	Y/Y	Yes
**5**	F	1	13	NSAID	No	Neg	Y/Y	No	Y/Y	Yes
**6**	F	1	15	NSAID	No	Pos	Y/Y	No	Y/Y	Yes
**7**	F	0	2	No	No	Pos	Y/Y	No	Y/Y	Yes
**8**	F	1	11	No	No	Neg	N/N	Yes	N/N	No
**9**	F	72^§^	8	No	No	Pos	Y/N	No	Y/N	Yes
**10**	F	120*	12.5	NSAID	No	Pos	Y/N	No	Y/N	No
**11**	F	49*	7.5	NSAID	No	Pos	Y/N	No	Y/N	Yes
**12**	M	132*	15	No	No	Pos	Y/Y	No	Y/Y	No
**13**	F	48*	16.5	No	Yes	Pos	Y/Y	No	Y/Y	Yes
**14**	F	88*	11	NSAID	No	Pos	Y/Y	No	Y/Y	Yes

*M/F* male/female, *N/A* no data, *Y* yes, *N* no

*Has been in long-term remission off treatment

^§^Has been in remission on medication

^+^Age at sample

### Sample preparation and isolation of monocytes

Synovial fluid cells were isolated by centrifugation (500 g, 10 min), resuspended in PBS, counted and adjusted to a concentration of 1 × 10^6^ cells/ml to be analyzed by flow cytometry (see below). In parallel, monocytes were isolated from blood (*n* = 5) and synovial fluid (*n* = 9) for RNA extraction. Details on monocyte isolation can be found in supplementary methods. Monocyte purity was assessed by flow cytometry and contaminating cells were consistently < 5%.

### Flow cytometry

Synovial fluid-derived cells and whole blood were analyzed by a panel of nine surface markers. Whole blood or PBS resuspended synovial fluid-derived cells were incubated with the following antibodies against specific markers: CD3, CD14, CD16, CD19, CD40, CD56, CD86, CD163, and CD206. The clones and fluorophores are found in supplementary material. All antibodies were obtained from BD Biosciences, except CD14 that was obtained from BioLegend (used in 1:50 dilution in PBS for anti-human CD3 and anti-human CD19, 1:200 dilution for the rest). PBS was used as a negative control. In vitro defined polarization markers were confirmed to be differentially expressed by polarized monocytes from healthy individuals using cytokine cocktails as described previously (supplementary figure 1) [12]. Incubated blood was lysed (TQ-prep with Immunoprep reagent system, Beckman Coulter). Cells were analyzed by the BD FacsCANTO II flow cytometer (BD Biosciences) and BD FacsDiva Software (Version 8, BD Biosciences). Paired blood samples were used to set gates for the synovial monocytes. Gating strategy applied can be found in supplementary figure 2.

### Quantitative RT-PCR

Total RNA was extracted from monocytes isolated from synovial fluid and circulating monocytes by the RNeasy Mini Kit (Qiagen). RNA quantity was determined by a NanoDrop 1000 (Thermo Scientific). Isolated RNA was stored at − 80 °C until use. Two hundred fifty nanograms of total RNA was converted to cDNA using the RT^2^ First-Strand Kit (Qiagen). A Custom RT^2^ Profiler PCR Array (Qiagen) was designed containing 28 M1- or M2-related genes (supplementary table 1) and analyzed according to the manufacturer’s instructions using the StepOnePlus real-time PCR system (Applied Biosystems). Ct values were extracted, normalized to the geometric mean of three housekeeping genes (beta-2-microglubilin, β actin, and Peptidylprolyl Isomerase A), and analyzed by the ∆∆Ct method. In patients where RNA from circulating monocytes was not available, the Ct values of their synovial fluid monocytes were normalized and compared to the geometric means of those patients where RNA from circulating monocytes were collected.

### Phagocytosis and ROS production

Phagocytosis and ROS production was assessed by PhagoTest™ and PhagoBurst™ (BD Biosciences), respectively, according to the manufacturer’s instructions. Stimulation of phagocytosis was performed using opsonized *E. coli* and ROS production using PMA*.* Heparinized whole blood and uncentrifuged synovial fluid were used for analyses on a BD FacsCANTO II.

### Culture and polarization of monocytes

The ability of synovial fluid to induce polarization was investigated by stimulation of cultured monocytes. Peripheral blood mononuclear cells (PBMCs) were isolated from the whole blood of healthy volunteers using LymphoPrep. Monocytes were further extracted using magnetic CD14 microbeads and MACS LS columns (Miltenyi). Cells were cultured in RPMI 1640 medium supplemented with 2.05 mM l-glutamine (Gibco Life Technologies) and 10% normal human serum (Sigma-Aldrich) overnight at 37 °C, 5% CO_2_. The next day, the medium was replaced with a fresh medium containing 10% synovial fluid or 10% plasma. Clotting of plasma was performed by incubation with 3 mM CaCl_2_ and 5 mM MgCl_2_ for 1 h at 37 °C, before adding to the medium. Cells were cultured for 48 h before collection by application of ice-cold PBS/0.5 mM EDTA buffer and careful pipetting. The cells were analyzed for CD16, CD163, CD40, PDL1, CD86, and CD206. Details on clones and fluorophores are found in supplementary material. All antibodies were diluted 1:100. Some patients lacked paired plasma samples. In these patients, the values of the synovial fluid polarized monocytes were compared to the median of the MFI values from plasma-induced monocytes. Statistics were calculated only in paired samples. BD FACS Canto II and BD FacsDiva were used for analysis.

### Cytokine production by in vitro polarized monocytes

Monocytes from healthy donors were isolated as described above and cultured overnight. The next day, the medium was replaced with a fresh medium containing 1 μl/ml BD GolgiPlug (BD Biosciences) and supplemented with 20% synovial fluid (*n* = 13) or 20% clotted plasma (see above) from healthy donors (*n* = 3). LPS (1 ng/ml, Invivogen) was used as a positive control. Cells were cultured for 6 h, detached, and fixated using BD fixation/permeabilization kit according to the manufacturer’s instructions. Cells were then analyzed for anti-TNF PE (MAb11), anti-IL-6 BV421 (MQ2-13A5), anti-IL-8 Alexa fluor 488 (E8N1), and anti-IL-10 APC (JES3-18F1) (all from BD), diluted 1:100. Cells were analyzed using BD FacsCANTO II and the BD FacsDiva Software.

### Cytometric bead array

Cytokine levels in synovial fluid (*n* = 13) and plasma (*n* = 9) was measured using the cytometric bead array (CBA) enhanced sensitivity flex set including nine cytokines (IL-1B, IL-4, IL-6, IL-8, IL-10, IL17A, IL-17F, IFNγ, and TNF, BD Biosciences) according to the manufacturer’s instructions. Plasma and synovial fluid samples were diluted 1:3 in dilution buffer. Samples were analyzed by BD FacsCANTO II. The cytokine concentrations were determined using standards and the FCAP software (BD Biosciences).

### Immunohistochemistry

Slides were baked at 60 °C for 1 h before deparaffinization and rehydration using xylene and a decaying ethanol series. Dako EnVision+ System-HRP (DAB) Kit (Agilent/Dako) was utilized for signal detection. Following rehydration and blocking of peroxidase activity, the slides were blocked using 5% BSA/PBS for 30 min. Slides were washed in PBS and incubated with 1:500 diluted mouse anti-human CD163 (clone: EDHu-1), 1:2000 with mouse anti-human CD14 (clone: 1H5D8), or 1:250 mouse anti-human CD68 (clone: Y1/82A) in 2% BSA/PBS overnight at 4 °C. Slides were then washed, dehydrated, and mounted using Pertex Mounting Medium (HistoLab). Images were gathered using a Zeiss Primo Vert microscope (Zeiss) equipped with AxioCam ERc (Zeiss). All antibodies showed similar staining, but CD163 provided the least amount of background and was thus used for further analysis.

### In situ hybridization

To further investigate the macrophage profile in the synovium, in situ hybridization was used to analyze cytokine mRNA expression in synovial biopsies [19]. The RNAscope® 2.5 HD Chromogenic Duplex Assay (Advanced Cell Diagnostics) was performed according to the manufacturer’s instructions. Two different probes were used, targeting TNF (blue) and IL-10 (red). For detailed information see supplementary material. Images were acquired using Zeiss microscope. Sections stained with one dot or less per ten cells were considered to be negative.

### Immunofluorescence

Immunofluorescence was utilized to investigate polarization in synovial tissue using the traditional markers CD40 and CD206. Slides were deparaffinized as described above. Antigen retrieval was performed using citrate buffer pH 6.0 followed by blocking with 5% BSA and 5% donkey serum (Abcam) for 30 min, RT. Slides were next incubated o/n at 4 °C with primary antibodies: 1:250 rabbit anti-human CD163 (clone: EPR19518, Abcam), 1:250 mouse anti-human CD206 (clone:22–130, Antibodies-Online), and 1:100 goat anti-human CD40 (clone:glu21-Arg193, R&D Systems). The following day, slides were washed with PBS and incubated for 1 h at RT with secondary antibodies: (all 1:200, donkey anti-mouse Alexa Fluor 568, donkey anti-rabbit Alexa Fluor 568, and donkey anti-goat Alexa Fluor 647 (all Abcam). Images were acquired using Zeiss microscope.

### Statistics

Values are displayed as the median and interquartile range (IQR) if not otherwise indicated. Paired samples were analyzed by the Wilcoxon’s matched-pairs signed-rank test. Statistical analyses were performed by Prism 7. *P* < 0.05 was considered statistically significant.

## Results

### Patient characteristics and data

In total, 14 patients fulfilled the set inclusion criteria; oligoarticular JIA, untreated (with the exception of NSAID), and undergoing a therapeutic arthrocentesis. Patients 1–8 were newly diagnosed whereas patients 9–14 had been diagnosed with oligoarticular JIA previously, but had been in stable remission without treatment for at least 6 months. Patient data and characteristics are summarized in Table 1. Two additional patients were primarily included as they met the inclusion criteria but were later found to have systemic-onset JIA and enthesitis-related JIA, respectively. They were thus excluded from the final compilation, but data from these patients are presented in the supplementary material.

### Synovial fluid monocytes from patients with oligoarticular JIA have altered CD14 and CD16 expression compared to circulating monocytes

**Fig. 1:**
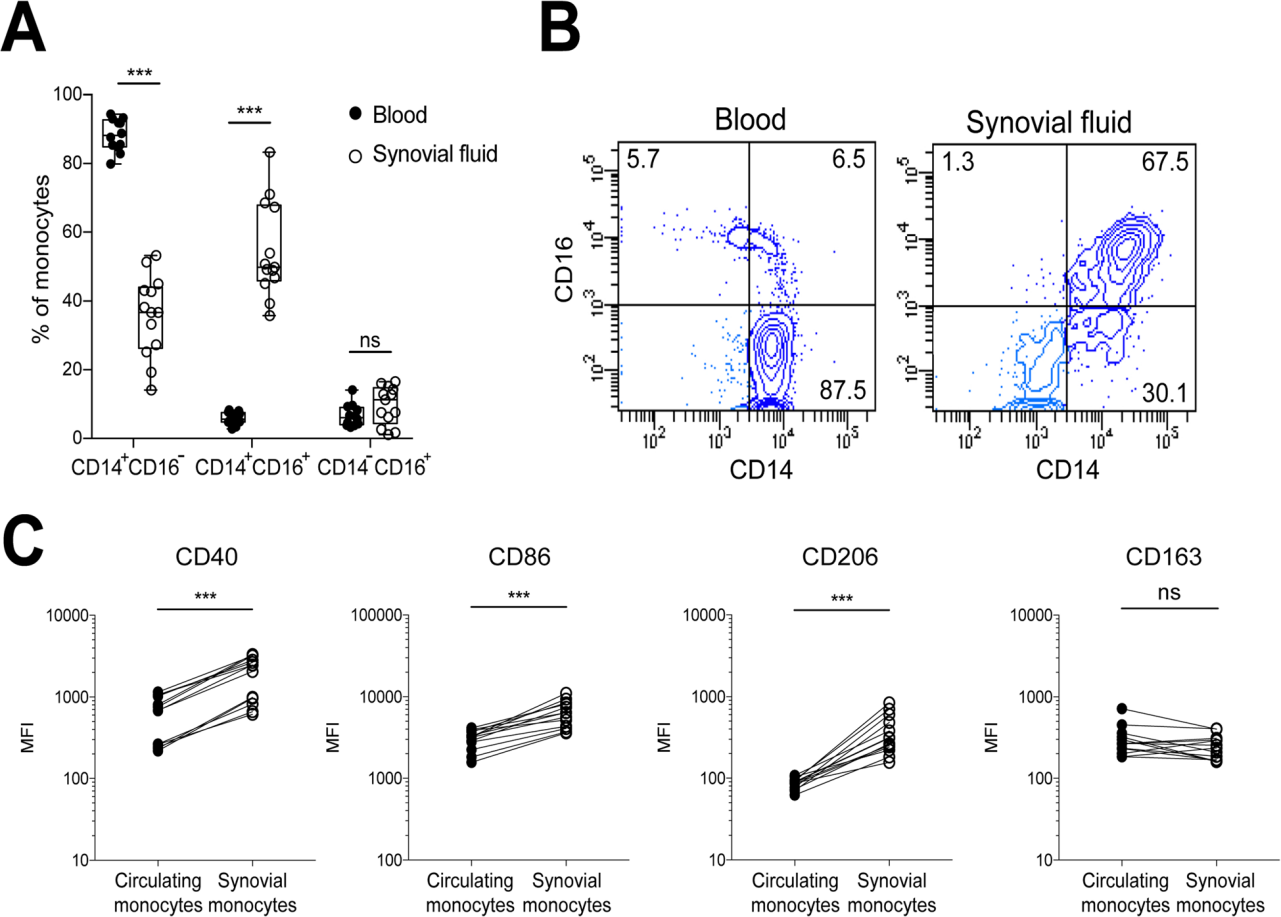
Synovial fluid monocytes express both M1- and M2-related polarization markers at the surface level. Synovial fluid and paired blood samples were collected from patients with oligoarticular JIA and analyzed by flow cytometry. **a** Monocyte subset distribution in the blood and synovial fluid based on CD14 and CD16 expression, *n* = 13, ***p < 0.001. **b** Representative gating plots based on CD14 and CD16 as summarized in **a**, for circulating- and synovial monocytes, respectively. Given that CD14-positive monocytes represent the majority of monocytes in synovial fluid, they were gated for further analysis. Compared to circulating monocytes, synovial monocytes expressed high levels of the **c** M1-like marker CD40 median (IQR): 2452 (895–3213) vs 688 (253–1066), CD86 6414 (4628–8328) vs 3247 (2561–3872), and the M2 marker CD206 310 (232–432) vs 88 (75–98). There was no difference in the expression of CD163 235 (168–303) vs 268 (217–406). Statistics was performed using Wilcoxon matched-pairs signed rank test (*n* = 13, ***p < 0.001). Data are shown as median. MFI-median fluorescence intensity

We first investigated if the distribution of monocyte subsets differs between synovial fluid and paired blood, based on CD14/CD16 expression. We observed a significant decrease in the frequency of CD14^+^CD16^−^ monocytes in synovial fluid compared to blood (median % positive cells): (36.7% vs 88.2%, *p* < 0.001) with a corresponding increased frequency of intermediate CD14^+^CD16^+^ monocytes (49.8% vs 5.7%, *p* < 0.001, Fig. 1a-b). There was no significant difference in the frequency of CD14^−^CD16^+^ monocytes. Accordingly, MFI values of both CD14 (20,441 vs 7849, *p* < 0.001) and CD16 (1736 vs 238, *p* < 0.001) were increased in the monocyte CD14^+^ population in synovial fluid compared to blood (Supplementary figure 3A).

### Synovial fluid monocytes have increased surface expression of CD40, CD206, and CD86 expression, but not of CD163

To explore synovial fluid monocyte polarization, paired synovial fluid and blood samples were analyzed for multiple surface markers. Synovial fluid monocytes from patients with oligoarticular JIA had increased expression of the M1 marker CD40 (2452 vs 688, *p* < 0.001), CD86 (6414 vs 3247, *p* < 0.001), and the M2 marker CD206 (310 vs 88, *p* < 0.001), as compared to patients’ circulating monocytes (Fig. 1c). CD40 was consequently higher expressed in synovial monocytes, but not all synovial fluid monocytes had higher expression of CD206 compared to circulating monocytes. Gating on percentage positive cells, barely 37% (median) of the synovial fluid monocytes expressed CD206, even though the MFI values were markedly increased. The CD206^+^ cells also expressed high levels of CD40 (Supplementary figure 3B). Intermediate CD14^+^CD16^+^ displayed higher expression of all markers compared to classical CD14^+^CD16^−^ monocytes (supplementary figure 3C). There was no significant difference in the expression of the M2 marker CD163 (235 vs 268). Synovial fluid monocytes from the two patients with systemic-onset JIA and enthesitis-related JIA, respectively, showed increased expression of CD163, as previously described (supplementary figure 3D). Finally, the expression of polarization markers in the circulating monocyte subsets was compared to the synovial fluid monocyte subsets, showing similar trends irrespectively of subsets (supplementary figure 4A-C). Thus, synovial fluid monocytes from children with oligoarticular JIA have a surface expression pattern of combined M1(IFNγ) and M2(IL-4), but not M2(IL-10).

### Synovial fluid monocytes display a similar and specific mixed M1(IFNγ)- and M2(IL-4)-like mRNA pattern

**Fig. 2:**
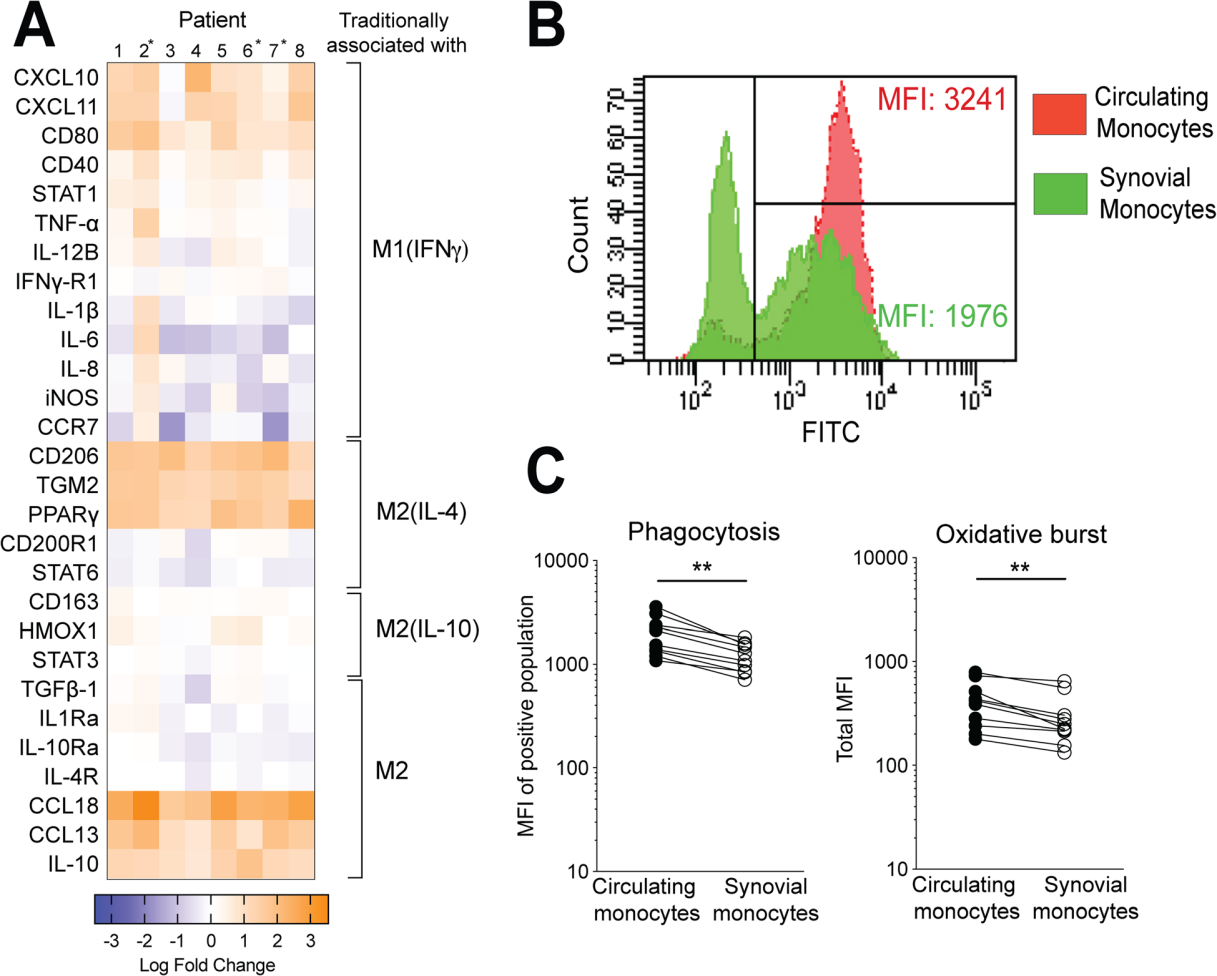
Synovial monocytes display a complex M1–M2-mRNA pattern and have an impaired phagocytosis. **a** Heatmap based on qPCR analysis of the fold change between synovial and circulating monocytes using 28 polarization-related markers at the surface to transcription level. Data was normalized using the ΔΔCt method to the geometric mean of the house-keeping genes PPIA, ACTB, and B2M. Log base 10. Paired *n* = 5, *patients analyzed without paired mRNA from circulating monocytes, *n* = 3. Unpaired data was analyzed based on the geometric mean of the patients with paired circulating monocytes. **b** Representative gating plot of the phagocytosis analysis were monocytes phagocytosed FITC labeled opsonized *E. coli*. The MFI of the positive population was used for further analysis. **c** Synovial monocytes display an impaired phagocytosis compared to circulating monocytes: 1082 (846–1702) vs 1462 (1237–2353), **p < 0.01, *n* = 9. Oxidative burst performance by synovial monocytes is affected using PMA as stimuli 234 (170–298) vs 337 (211–432), **p < 0.01, *n* = 9. Statistics was performed using Wilcoxon matched-pairs signed rank test. Data are shown as median. MFI-median fluorescence intensity

To more thoroughly investigate the polarization pattern of synovial monocytes, monocytes were analyzed at the mRNA level for 28 polarization-related genes using qPCR. In accordance with flow cytometry data, synovial fluid monocytes expressed both M1(IFNγ)- and M2(IL-4)-related markers, a result showing high similarity across patients (Fig. 2a). In total, 14 out of 28 genes showed a more than 2-fold change in expression compared to circulating monocytes, including cytokines, chemokines, enzymes, and transcription factors. M2(IL-10) in vitro defined markers CD163, HO-1, and STAT3, were not differentially expressed between the synovial fluid and circulating monocytes. Neither were other, well-defined markers of both M1 and M2 polarization, e.g., iNOS and CD200R1, increased in synovial fluid monocytes. Taken together, these data demonstrate a polarization and activation pattern in synovial fluid monocytes that is not exclusively M1- or M2-like. Instead, there is a specific pattern combined of traditional in vitro defined M1(IFNγ) (CD80, STAT1, and CXCL10) and M2(IL-4) (CD206, CCL18, and PPARγ), but not M2(IL-10)-related markers across all patients.

### Synovial fluid monocytes have reduced phagocytosis and oxidative burst

Next, we sought to determine if synovial fluid monocytes are altered at the functional level, assessing phagocytosis and oxidative burst. Synovial fluid monocytes showed a reduced ability to phagocytose as compared to paired circulating monocytes (MFI; 1082 vs 1462, *p* < 0.01), Fig. 2b, c) as well as being less prone to undergo oxidative burst upon stimulation with PMA (MFI; 234 vs 337, *p* < 0.01, Fig. 2c). This was not an effect of synovial fluid alone, as the addition of cell-free synovial fluid to the whole blood at a 10:1 ratio did not result in altered phagocytosis (data not shown).

### Synovial fluid from patients with oligoarticular JIA induces upregulation of some M2-, but not M1-like markers on healthy monocytes

**Fig. 3:**
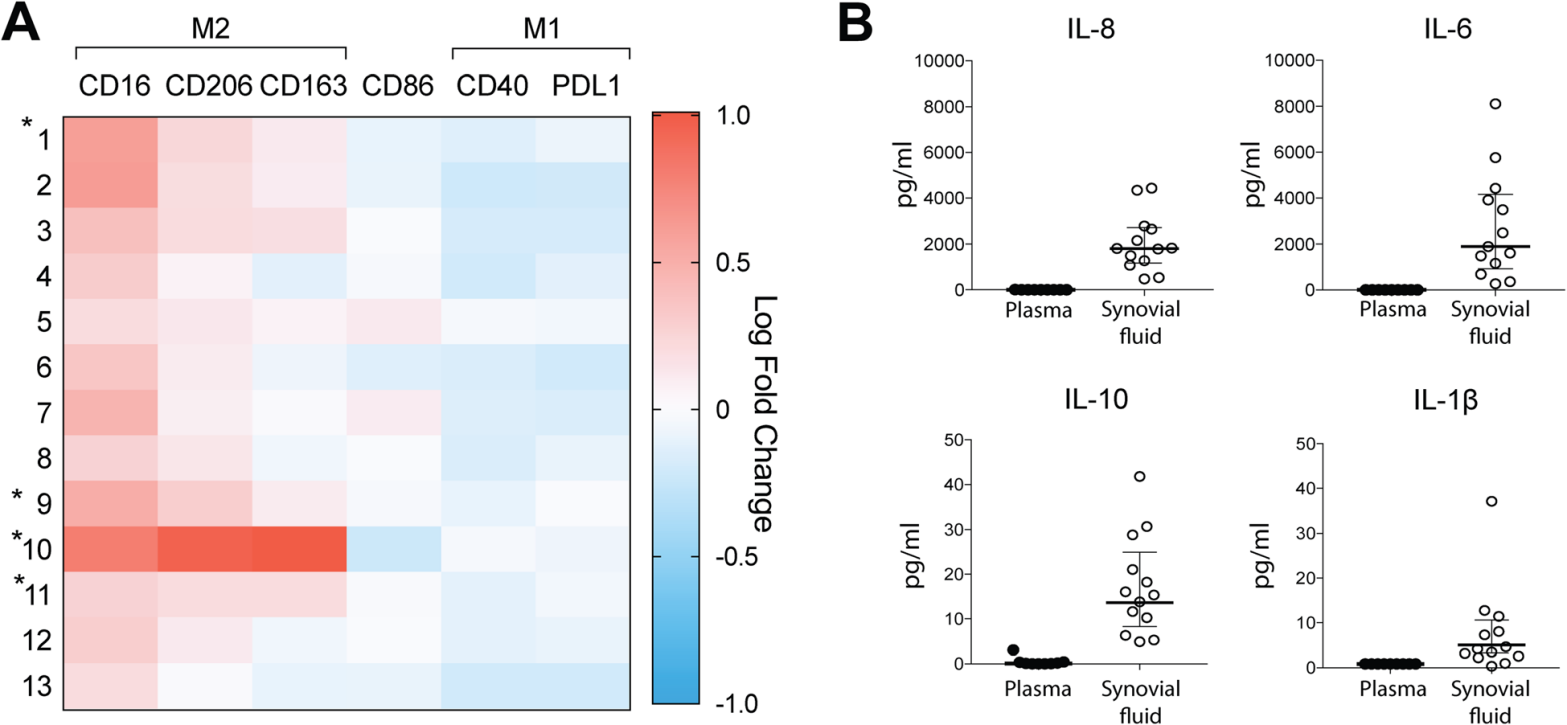
Synovial fluid alone does not induce the observed in vivo polarization pattern. **a** Paired SF and plasma samples were used to induce polarization in in vitro cultured monocytes from healthy donors and analyzed by flow cytometry. The fold change of the markers between SF and plasma was calculated. SF induced an M2-like pattern as compared to paired plasma, with a significant MFI fold change in all markers, except CD163. *Unpaired SF samples. Data from these patients was calculated against the geometric mean of the patients with plasma samples (paired SF and plasma, *n* = 9; unpaired SF, *n* = 4). **b** Cytokines in the synovial fluid were analyzed by a cytometric bead assay and contained measurable levels of IL-1β, IL-6, IL-8, and IL-10 compared to paired plasma that contained none to very low detectable levels. SF *n* = 13, plasma *n* = 9. Statistics was performed on paired data using Wilcoxon matched-pair signed rank test. Data are shown as median and interquartile range. MFI-median fluorescence intensity. SF: synovial fluid

To determine if the synovial fluid is responsible for the observed polarization pattern, monocytes from healthy donors were cultured in vitro and polarized using synovial fluid or plasma from JIA patients. Synovial fluid significantly upregulated M2 markers CD16 and CD206 (*p* < 0.05), but not CD163 (*p* = 0.2) as compared to plasma polarized monocytes (Fig. 3a). Further, synovial fluid downregulated M1(IFNγ)-marker CD40 and the activation marker CD86 (*p* < 0.05). Thus, synovial fluid alone did not induce the specific pattern seen in patients’ synovial fluid monocytes.

### Synovial fluid did not induce cytokine production in healthy monocytes

To further elucidate the inflammatory role of synovial fluid, cell-free synovial fluid (*n* = 13) or plasma from healthy controls (*n* = 3) was used to activate cytokine production in monocytes from healthy donors. The synovial fluid did not induce any production of pro- or anti-inflammatory cytokines TNF, IL-6, IL-8, or IL-10 compared to plasma from healthy donors, whilst LPS clearly induced the production of most cytokines (Supplementary figure 5).

### Cytokine pattern in oligoarticular JIA patients’ synovial fluid

Next, we explored the cytokine environment within the synovial fluid (*n* = 13) considering several inflammatory cytokines: IL-1β, IL-4, IL-6, IL-8, IL-10, IL-12p70, IL-17A, TNF, and IFNγ (Fig. 3b). We found measurable concentrations (median (IQR) of IL-1β (3.9 pg/ml (2.2–8.9)), IL-6 (1755 pg/ml (646–4038)), IL-8 (1801 pg/ml (941–2696)), and IL-10 (14.6 pg/ml (9.3–23)) in the synovial fluid. Interestingly, traditional cytokines used to induce M1(IFNγ) and M2(IL-4) were not present at measurable concentrations in the synovial fluid. Furthermore, we found very low or no measurable levels of IL-12p70, TNF, and IL-17A (Supplementary figure 6). Concentrations of the respective cytokines were low or not measurable in plasma.

### Macrophages are prominent in the synovial membrane of patients with oligoarticular JIA and show a mixed polarization pattern at both mRNA and surface level

**Fig. 4:**
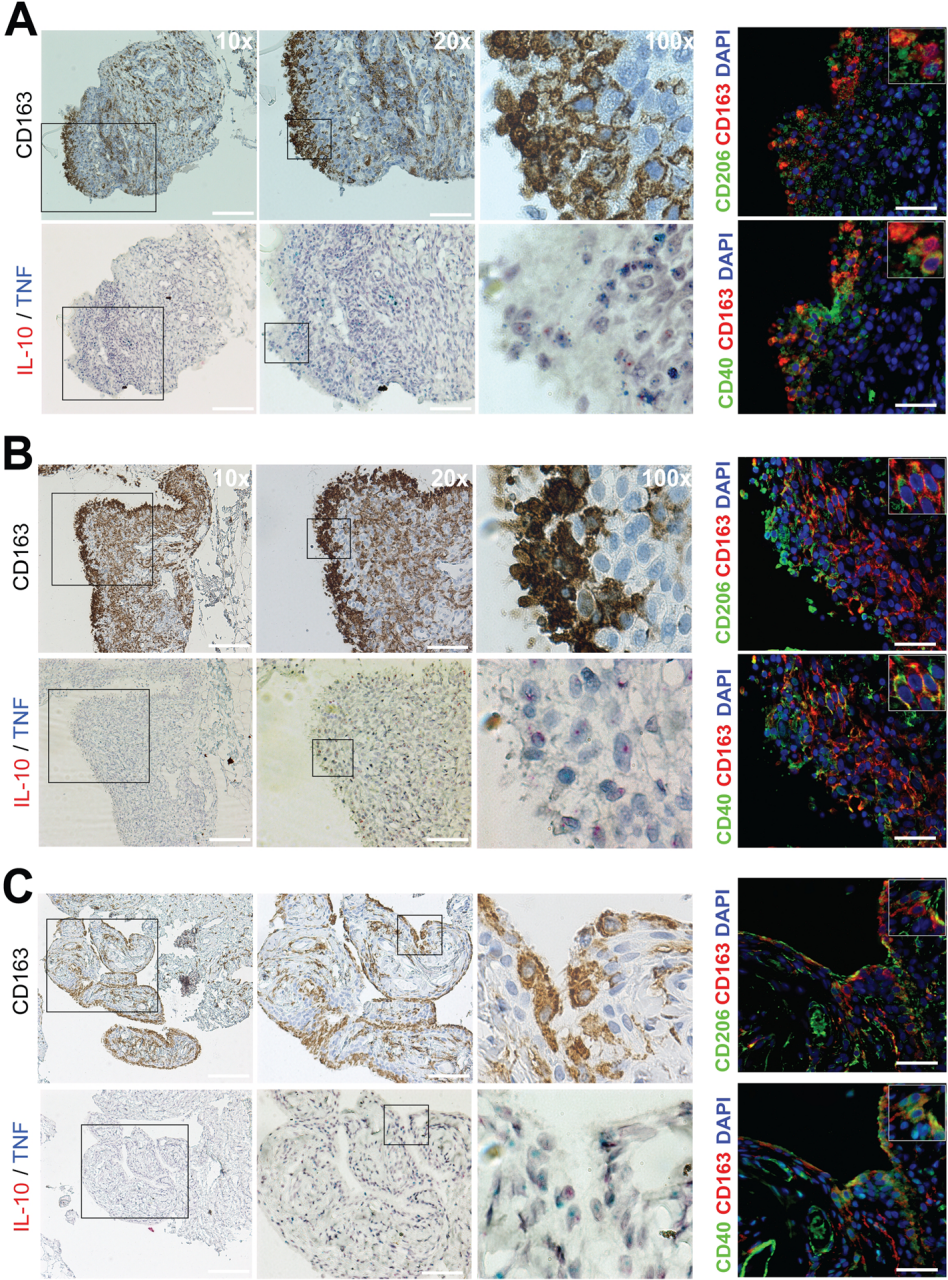
Macrophages are present in the lining- and sub-lining layer of the synovial membrane and co-localize with IL-10 and TNF mRNA. **a**–**c** Representative images of **a** patient 1, **b** patient 3, and **c** patient 8. Even if we could not detect an increased expression of CD163 in synovial fluid monocytes, it is clearly present in tissue monocytes/macrophages. Antibodies to CD14 and CD68 were also tested and provided similar staining, but CD163 provided the most specific staining. Monocytes and macrophages are found extensively in both the lining and sub-lining region of the synovial membrane*.* In situ hybridization targeting IL-10 (red dots) and TNF (blue dots) mRNA was performed in subsequent sections. Both TNF and IL-10 are found in areas that are dense in macrophage numbers. Staining for polarization markers revealed both single and co-expression of CD40 and CD206 in macrophages, as well as in other cell types as neither CD40 nor CD206 is specific for monocytes/macrophages. Individual channel images are found in grayscale in supplementary Fig. 7. Scale bar in 10x: 220 μm and in 20×: 100 μm. For immunofluorescence: 50 μm

Finally, to explore the polarization of monocytes/macrophages in the synovial tissue of patients with JIA, biopsies (*n* = 3) were collected and stained for polarization markers and cytokine mRNA. Using immunohistochemistry, macrophages were identified in both the lining and sub-lining region (Fig. 4) of the synovial membrane in all patients. The subsequent sections were stained by in situ hybridization for IL-10 (red dots) and TNF (blue dots) mRNA as IL-10 is traditionally considered to be produced by M2(IL4 and IL10) macrophages and TNF by M1(IFNγ) macrophages. Both IL-10 and TNF mRNA can be seen in the lining and the sub-lining region (Fig. 4). Controls of the experiment can be found in supplementary figure 7. Finally, both single and co-expression of the markers CD40 and CD206 were seen in monocytes/macrophages using immunofluorescence (Fig. 4). Single-channel images are available in grayscale in supplementary figure 7 A-C. Thus, monocytes/macrophages express a mixed M1/M2 pattern both at the surface and mRNA level consistent with the pattern demonstrated in synovial fluid.

## Discussion

In this study, we show that children with oligoarticular JIA have a distinct activation- and polarization pattern in synovial fluid monocytes. Monocytes displayed features of a mixed M1(IFNγ)/M2(IL-4), but not M2(IL-10)-like phenotype, both at the surface and at the mRNA level. Functionally, these cells had reduced phagocytosis compared to circulating monocytes. Synovial fluid alone induced an M2-like pattern compared to paired plasma. The synovial membrane contained dense populations of lining- and sub-lining macrophages, with a mixed expression of IL-10, TNF, CD40, and CD206. Thus, polarization occurs both in the synovial tissue and fluid. Taken together, our data indicate important roles for monocytes and macrophages and suggest a distinct pattern of immune activation in oligoarticular JIA.

In arthritis, monocytes produce pro-inflammatory cytokines, angiogenetic factors, and display increased levels of markers involved in antigen presentation. In this study of children with oligoarticular JIA, we observed an increased frequency of synovial CD14^+^CD16^+^ monocytes. Synovial CD14^+^CD16^+^ monocytes have also been shown to be expanded in rheumatoid arthritis (RA) and osteoarthritis (OA) and to promote both Th1 and Th17 responses in vitro [6, 20]. We could see an induction of CD16 in cultured monocytes following polarization with synovial fluid. The synovial fluid contained high levels of IL-10, a potent inducer of CD16 expression in monocytes in vitro [21]. Given that we observe a similar pattern across monocyte subsets, the increased CD16 expression in monocytes is likely due to the influence of the local environment in the synovium and synovial fluid, and most probably does not reflect specific recruitment of intermediate CD14^+^CD16^+^ monocytes from the circulation.

Polarization alters cellular functions, such as the ability to phagocytose, as well as the inflammatory impact the cells have on their environment. Polarization has been investigated for its importance in several settings, including obesity, cancer, and arthritis [13]. However, the polarization patterns and its consequences have not been studied in children with oligoarticular JIA. Here, we identified mRNA signatures of both M1(IFNγ) and M2(IL-4) in vitro defined markers [8, 11, 22]. Indeed, several M1(IFNγ)- and M2(IL-4)-related chemokines (e.g., CXCL10/11 and CCL13/18), cytokines (e.g., TNF and IL-10), and receptors (e.g., CD80 and CD206) were upregulated but not other traditional markers (e.g., iNOS and CD200R1). Multiple cytokines and chemokines have previously been shown to be elevated at the protein level in patients with oligoarticular JIA, e.g., CXCL10, IL-6, and IL-8 [23]. This is in line with our cytokine analysis, suggesting IL-6 and IL-8 to be the most prominent cytokines in the synovial fluid. Thus, the inflammatory pattern of the children with oligoarticular JIA could not be fitted into a traditional polarization pattern, but should rather be considered to be its own specific one.

Interestingly, we could not detect any increase in CD163 neither on the surface- nor at mRNA level in our oligoarticular JIA patients, but has previously been observed in enthesitis-related JIA [16, 17]. In our study, two patients with systemic-onset JIA and enthesitis-related JIA, respectively, were initially included as they presented with the oligoarticular disease. In these patients, we observed an increased surface expression of CD163 in synovial fluid monocytes compared to monocytes from oligoarticular patients. In addition, CD163 has been linked to several adult arthritides, including increased expression in RA synovial fluid monocytes, and following stimulation of monocyte-derived macrophages with synovial fluid from SpA patients [15, 24]. M2(IL-10) -like macrophages correlates with inflammatory features in SpA [25, 26]. M1/M2 imbalance has been linked to osteoclastogenesis in RA, to disease severity in OA, and a mixed expression of CD206/CD163 in circulating monocytes from patients with systemic JIA [27–29]. Thus, although an M1/M2 imbalance has been demonstrated in arthritides, the children with oligoarticular JIA in our study show a specific pattern that, at least partly, is distinct from what is described in other forms of adult and juvenile arthritides. This needs further investigations, but might suggest that different pathophysiological mechanisms drive inflammation in oligoarticular JIA.

In addition to having a distinct activation and polarization pattern, our patient group of oligoarticular JIA patients shows consistent homogeneity throughout the different experiments. This was regardless of whether the patients were newly diagnosed or had been in stable remission without treatment. Thus, the monocyte/macrophage activation pattern was not dependent on disease duration, but rather on disease presentation. All included patients share a similar presentation of active disease, being either treatment naïve or off treatment for at least 6 months before inclusion. A similar pattern seen in all children strengthens the hypothesis that this form of JIA shares a common pathogenesis.

Synovial fluid from the JIA patients induced a mixed M2(IL-4)/M2(IL-10)-like phenotype in monocytes from healthy individuals even though we did not detect any M2(IL-10)-monocytes in the patients. We could not detect any de novo production of IL-6, IL-8, or IL-10 from monocytes stimulated with synovial fluid, even if these cytokines were found in the synovial fluid [23]. Taken together, this indicates that the polarization pattern detected in the patients is not only a result of the inflammatory milieu of the synovial fluid. We therefore hypothesized that the monocytes might attain, at least partly, their polarization pattern when migrating through the synovial tissue. In synovial tissue, we identified macrophages/monocytes in both the lining and sub-lining regions, an observation that has been previously seen in RA and SpA [14]. Additionally, a general expression of TNF and IL-10 mRNA in macrophage rich areas in synovial biopsies, as well as single and co-expression of CD40 and CD206 in monocytes/macrophages supports the idea that the macrophages/monocytes are polarized, suggesting that M1 (and M2) features can be obtained in the synovial membrane, and not exclusively in the synovial fluid. The macrophages have been shown to produce different levels of pro- and anti-inflammatory mediators in different diseases, and their number correlates to disease activity in RA [14, 30, 31]. Although we did not perform an in-depth analysis of the macrophage polarization pattern and are limited by the sample size, our data suggests that macrophages are present and activated within the synovium, with both pro- and anti-inflammatory features, in patients with oligoarticular JIA.

We also investigated if the synovial monocytes were functionally affected with regard to phagocytosis and oxidative burst. We observed a reduced ability of synovial monocytes to phagocytose and to perform oxidative burst by reactive oxygen species (ROS) production in response to PMA. Defective oxidative burst, due to a variation in the *ncf1* gene, has been shown to result in an increased severity in T cell-dependent arthritis in animal models, an observation counteracted by a specific restoration of ROS production in macrophages [32]. Decreased phagocytosis has been associated with M1(IFNγ) polarization. Conversely, unpolarized and M2-polarized cells displayed an unaltered or increased phagocytosis [10, 33]. These studies suggest that impaired ROS production and phagocytosis by monocytes and macrophages contribute to inflammation. However, more research is needed to elucidate the role of ROS and phagocytosis in oligoarticular JIA.

Limitations to our study include some methodological issues. We were not able to sort different monocyte populations. It would be of interest to sort CD206-positive and CD206-negative cells and investigate their mRNA expression and cytokine pattern separately. Additional limitations concern the activity of signaling pathways, i.e., at the protein and phosphorylation level, where the JAK/STAT pathway is not necessarily reflected at the mRNA level. Further research should be conducted regarding the functional role of these cells within the joint as recent studies have suggested a protective role of tissue-resident synovial monocytes/macrophages [34]. Finally, due to their importance in arthritis, further investigation of the synovial tissue macrophage polarization pattern in a larger sample size is of interest to determine their role in the pathogenesis.

## Conclusion

We observed a specific synovial monocyte polarization pattern in children with untreated oligoarticular JIA. The synovial fluid monocytes were functionally affected as shown by reduced phagocytosis and oxidative burst. We could also detect polarized macrophages in the synovial membrane of the children with the production of both pro- and anti-inflammatory cytokines. In addition, the children displayed a distinct homology in their monocyte pattern regardless of disease duration, suggesting common pathophysiological mechanisms of arthritis in this patient group. Further studies on these cells and functional implications in the pathogenesis provide an interesting area to explore for future treatments.

## Supplementary information

**Additional file 1: Supplementary methods**. **Supplementary table 1.** List of target genes analyzed in monocytes by RT-qPCR.

**Additional file 2: Supplementary figure 1.** Expression of surface markers in different monocyte phenotypes. Monocytes were isolated from healthy controls. These were then stimulated with cytokine cocktails to induce specific phenotypes: M1(IFNγ)- IFNγ (10 ng/ml) and LPS (10 ng/ml), M2(IL-4)- IL-4 (20 ng/ml) and IL-13 (25 ng/ml), and M2(IL-10)- IL-10 (25 ng/ml) and Dexamethasone (10 nM). As a result, we observed a clear upregulation of specific markers in different phenotypes following polarization. This has also been tested individually for IFNγ, IL-4 and IL-10, respectively.

**Additional file 3: Supplementary figure 2**. Strategy for investigation of polarization. Gating strategy applied to investigate the distribution of surface markers in circulating or synovial fluid monocytes.

**Additional file 4: Supplementary figure 3**. Further characterization of monocyte polarization by flow cytometry. (A) MFI values of the markers CD14 and CD16. (B) Shows a representative gating plot and the percentage of CD206 positive synovial monocytes as well as the CD40 expression of the CD206 positive and negative cells, respectively. The gates were set based on paired circulating monocytes. (C) MFI of the polarization markers in classical and intermediate synovial monocytes (D) MFI of the polarization related markers of patients with oligoarticular JIA, and two patients with enthesitis-related JIA and systemic-onset JIA, respectively. Statistics were performed using Wilcoxon matched-pairs signed rank test, **p < 0.01, ***p < 0.001.

**Additional file 5: Supplementary figure 4**. Expression of polarization markers across monocyte subsets. Shows the MFI values of each investigated polarization markers in circulating vs synovial populations of (A) CD14^+^CD16^−^ ‘classical’ monocytes (B) CD14^+^CD16^+^ ‘intermediate’ monocytes and (C) C14^−^CD16^+^ ‘alternative’ monocytes.

**Additional file 6: Supplementary figure 5**. Activation of cytokine production in healthy monocytes by synovial fluid. Monocytes from healthy donors were stimulated with 20% synovial fluid or 20% plasma from healthy controls. Synovial fluid did not induce production of IL-6, IL-8, IL-10 or TNF in healthy monocytes as compared to plasma from healthy donors. LPS (1 ng/ml) was used as positive control. SF *n* = 13, plasma *n* = 3.

**Additional file 7: Supplementary figure 6**. Additional cytokine measurements in patients with oligoarticular JIA. Cytokines were measured in synovial fluid and plasma from patients with oligoarticular JIA. The cytokines concentration of IL-17A, IL-4, IFNγ, IL-12 and TNF in plasma were below detection limit in most of the patients.

**Additional file 8: Supplementary figure 7**. Grayscale images of the investigated polarization markers and in situ hybridization controls. (A-C) individual channels in grayscale of CD163, CD206, CD40 and DAPI in the three synovial biopsies analyzed (patient 1, 3 and 8, respectively). (D) Representative images of positive and negative controls of the in situ hybridization experiments. Scale bar: 50 μm.

## Data Availability

Anonymized datasets used and/or analyzed during the current study are available from the corresponding author on reasonable request.

## Abbreviations

ANA: Anti-nuclear antibodies
IL: Interleukin
IFN: Interferon
JIA: Juvenile idiopathic arthritis
LPS: Lipopolysaccharide
MFI: Median fluorescence intensity
NSAID: Non-steroid anti-inflammatory drug
OA: Osteoarthritis
PBMC: Peripheral blood mononuclear cells
RA: Rheumatoid arthritis
ROS: Reactive oxygen species
SpA: Spondylarthritis
TNF: Tumor necrosis factor-alpha
