# Supplementary material for: Children with oligoarticular juvenile idiopathic arthritis have skewed synovial monocyte polarization pattern with functional impairment—a distinct inflammatory pattern for oligoarticular juvenile arthritis
Source: Arthritis Res Ther. 2020 Aug 12;22:186. doi: 10.1186/s13075-020-02279-9 (PMC7425414; doi:10.1186/s13075-020-02279-9)
Supplement: Supplementary file 1 — Additional file 1: Supplementary methods. Supplementary table 1. List of target genes analyzed in monocytes by RT-qPCR. [file 13075_2020_2279_MOESM1_ESM.docx]

**SUPPLEMENTARY MATERIAL**

**Supplementary methods**

**Patient data, materials and sample collection**

Peripheral blood (n=13) was collected with 2.7ml vacutainer tubes containing EDTA (1.8mg/ml, BD Biosciences) and heparin tubes (BD Biosciences). EDTA treated blood was used for flow cytometry and heparinized blood was used for phagocytosis and monocyte isolation experiments. EDTA treated blood was centrifuged at 1900g for 10min and the plasma was collected and stored at -80°C. Freshly isolated synovial fluid was centrifuged at 500g for 10min, and the supernatant was stored at -80°C. Biopsies were fixated in 4% formaldehyde for 24hrs and dehydrated in ethanol followed by paraffin embedment. Paraffin blocks were sectioned into 3μm thick slices and mounted on SuperFrost glass sections (Thermo Scientific).

**Sample preparation and isolation of monocytes**

Monocytes were isolated from heparinized blood or synovial fluid. For blood, peripheral mononuclear cells (PBMCs) were isolated by density gradient (LymphoPrep, Alere Technologies) and monocytes were further isolated by a two-step magnetic bead sorting by first incubating with anti-human biotin-conjugated CD14 (diluted 1:125 in PBS, clone: MEM-15, Thermo Scientific) followed by a PBS wash and incubation with streptavidin-conjugated dynabeads (0.4μg/ml, Invitrogen). Coupled cells were placed in a magnetic holder (Thermo Scientific) and repeatedly washed three times. For synovial cells, resuspended cells were adjusted to 8x10^6^ cells/ml and incubated with anti-human biotin-conjugated CD66b diluted 1:62.5 in PBS (clone: G10F5, Biolegend) and dynabeads (0.6μg/ml) to separate the CD66b^+^ cells. Next, the supernatant was transferred, and monocytes were isolated by anti-human CD14 and dynabeads as described above.

**Flow cytometry**

The following clones and fluorophores were used for flow cytometric analysis: anti-CD14 (clone: HCD14, BDV421), anti-CD40 (clone: 5C3, BDV510), anti-CD3 (clone: HIT3a, FITC), anti-CD19 (clone: HIB19, FITC), anti-CD56 (clone: B159, FITC), anti-CD163 (clone: GHI/61, PE), anti-CD206 (clone: 19.2, APC), anti-CD86 (clone: 2331 (FUN-1), PECy7) and anti-CD16 (clone: 3G8, APCH7).

**Culture and polarization of monocytes**

The following clones and fluorophores were used for flow cytometric analysis: PE anti-CD163 (clone: GHI/61, BD Biosciences), FITC anti-CD86 (clone:FUN-1, Biolegend), APC-H7 anti-CD16 (Clone: 3G8, BD), BV510 anti-CD40 (Clone: 5C3, Biolegend), BV421 anti-CD274/PDL-1 (clone: MIH1, BD) and APC anti-CD206 (clone:19.2, BD).

***In situ* hybridization**

Two different probes were used, targeting TNF (Cat No: 310421, ref sequence: NM_000594.3) mRNA in channel 1 (C1) (green/blue) and IL-10 (Cat No: 602051-C2, ref sequence: NM_0005722) mRNA in C2 (red). In parallel, a PPIB(C2)/POLR2A(C1) probe and a bacterial RTU (C1 and C2) probe were used as positive and negative control, respectively (all probes from Advanced Cell Diagnostics). Sections were counterstained with hematoxylin (Vector Labs) and mounted using VectaMount Permanent Mounting Medium (vector Labs).

**Supplementary table 1:** list of target genes analyzed in monocytes by RT-qPCR

| **Nr** | **Symbol** | **Full name** | **GenBank** |
| --- | --- | --- | --- |
| **1** | IL1B | Interleukin 1, beta | NM_000576 |
| **2** | IL1RN | Interleukin 1 receptor antagonist | NM_000577 |
| **3** | IL6 | Interleukin 6 | NM_000600 |
| **4** | IL8 | Intereukin 8 | NM_000584 |
| **5** | IL10 | Interleukin 10 | NM_000572 |
| **6** | CXCL10 | C-X-C Motif Chemokine 10 | NM_001565 |
| **7** | CXCL11 | C-X-C Motif Chemokine 11 | NM_005409 |
| **8** | CCL18 | C-C Motif Chemokine 18 | NM_002988 |
| **9** | CCL13 | C-C Motif Chemokine 13 | NM_005408 |
| **10** | CCR7 | C-C Motif Chemokine Receptor 7 | NM_001838 |
| **11** | IFN𝛾 | Interferon, gamma | NM_000619 |
| **12** | TNF⍺ | Tumor Necrosis Factor | NM_000594 |
| **13** | TGFB1 | Transforming Growth Factor-beta 1 | NM_000660 |
| **14** | CD80 | CD80/B7-1 | NM_005191 |
| **15** | CD200R1 | CD200 Receptor R | NM_138806 |
| **16** | CD206 | Mannose Receptor, MRC1 | NM_002438 |
| **17** | CD163 | Scavenger Receptor | NM_004244 |
| **18** | CD40 | Co-stimulatory Receptor | NM_001250 |
| **19** | HMOX1 | Heme Oxygenase 1 | NM_002133 |
| **20** | TGM2 | Transglutaminase 2 | NM_004613 |
| **21** | NOS2 | Nitric Oxide Synthase (iNOS) | NM_000625 |
| **22** | STAT1 | Signal Transducer and Activator of Transcription 1 | NM_007315 |
| **23** | STAT3 | Signal Transducer and Activator of Transcription 3 | NM_003150 |
| **24** | STAT6 | Signal Transducer and Activator of Transcription 6 | NM_003153 |
| **25** | IFNyR1 | Interferon gamma receptor 1 | NM_000416 |
| **26** | PPARγ | Peroxisome proliferator-activated receptor γ | NM_005037 |
| **27** | IL10RA | IL10 receptor subunit alpha | NM_001558 |
| **28** | IL4R | Interleukin 4 receptor 1 (alpha) | NM_000418 |
| **HK** | PPIA | Peptidylprolyl Isomerase A | NM_021130 |
| **HK** | ACTB | Actin, beta | NM_001101 |
| **HK** | B2M | Beta-2-microglobulin | NM_004048 |
|  | HGDC | Human Genomic DNA Contamination Control | NA |
|  | RTC | Reverse Transcription Control | NA |
|  | PPC | Positive PCR Control | NA |
