# Supplementary figures and images for: Children with oligoarticular juvenile idiopathic arthritis have skewed synovial monocyte polarization pattern with functional impairment—a distinct inflammatory pattern for oligoarticular juvenile arthritis
Source: Arthritis Res Ther. 2020 Aug 12;22:186. doi: 10.1186/s13075-020-02279-9 (PMC7425414; doi:10.1186/s13075-020-02279-9)

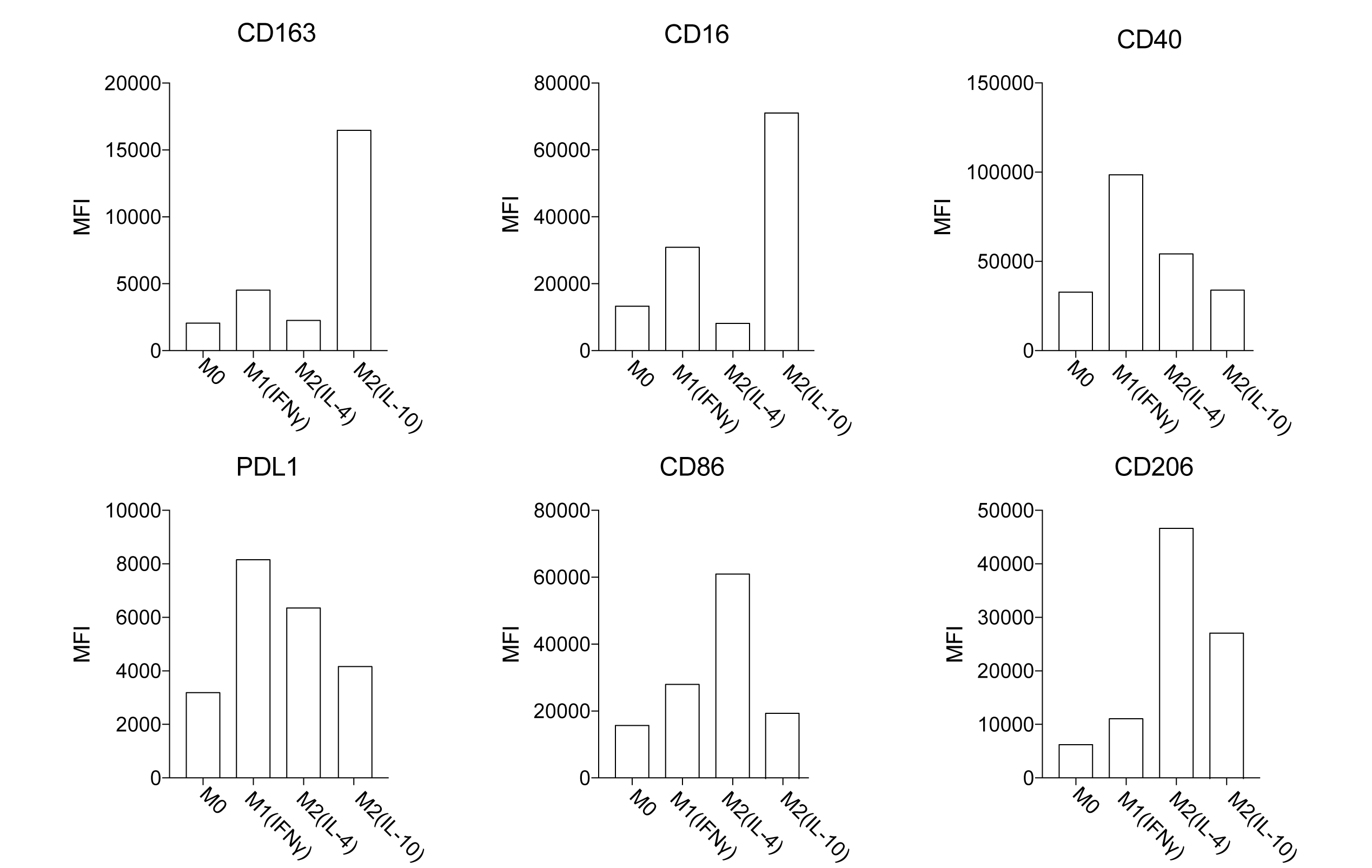

Supplement: Supplementary file 2 — Additional file 2: Supplementary figure 1. Expression of surface markers in different monocyte phenotypes. Monocytes were isolated from healthy controls. These were then stimulated with cytokine cocktails to induce specific phenotypes: M1(IFNγ)- IFNγ (10 ng/ml) and LPS (10 ng/ml), M2(IL-4)- IL-4 (20 ng/ml) and IL-13 (25 ng/ml), and M2(IL-10)- IL-10 (25 ng/ml) and Dexamethasone (10 nM). As a result, we observed a clear upregulation of specific markers in different phenotypes following polarization. This has also been tested individually for IFNγ, IL-4 and IL-10, respectively. [file 13075_2020_2279_MOESM2_ESM.tif]

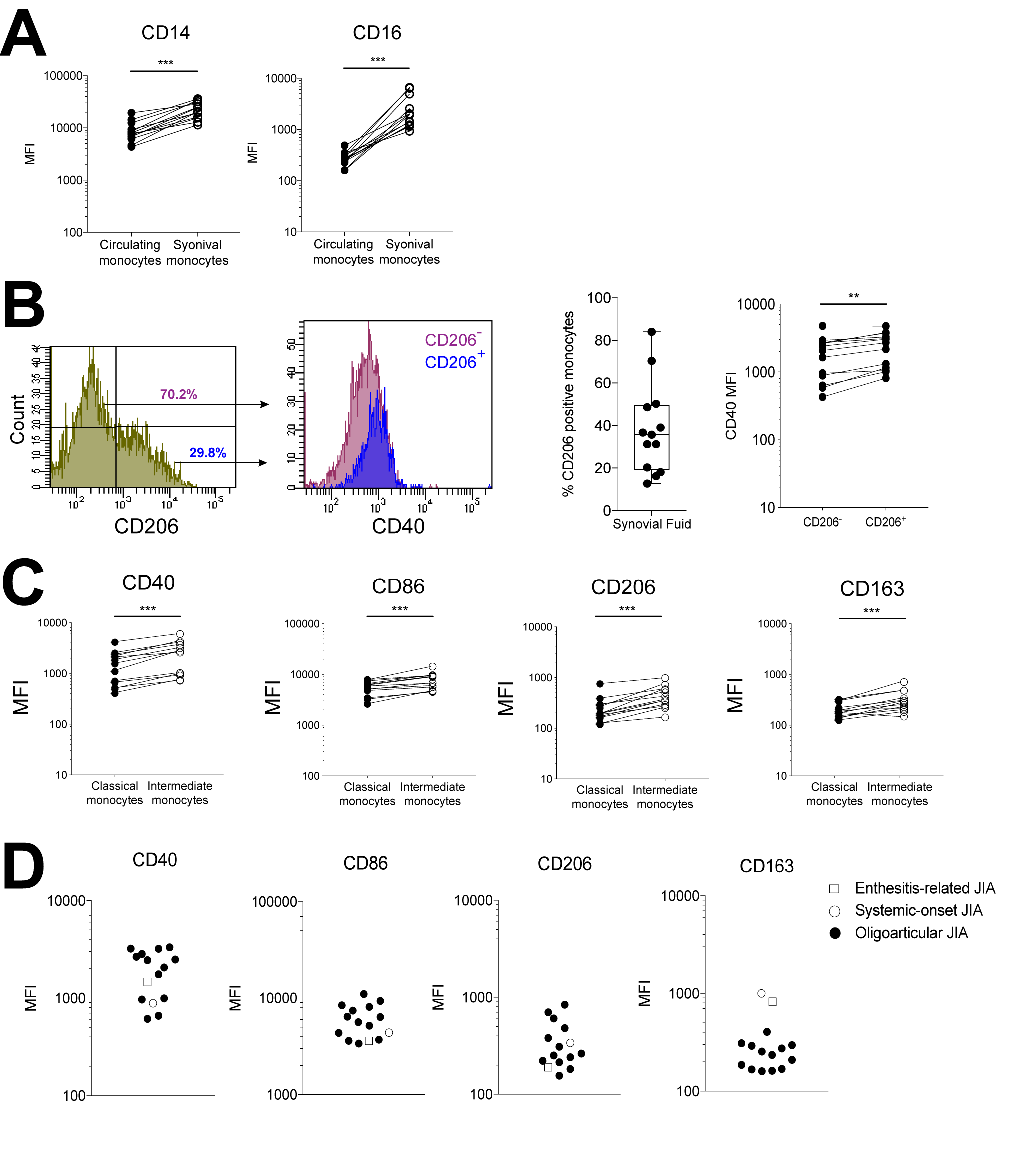

Supplement: Supplementary file 4 — Additional file 4: Supplementary figure 3. Further characterization of monocyte polarization by flow cytometry. (A) MFI values of the markers CD14 and CD16. (B) Shows a representative gating plot and the percentage of CD206 positive synovial monocytes as well as the CD40 expression of the CD206 positive and negative cells, respectively. The gates were set based on paired circulating monocytes. (C) MFI of the polarization markers in classical and intermediate synovial monocytes (D) MFI of the polarization related markers of patients with oligoarticular JIA, and two patients with enthesitis-related JIA and systemic-onset JIA, respectively. Statistics were performed using Wilcoxon matched-pairs signed rank test, **p < 0.01, ***p < 0.001. [file 13075_2020_2279_MOESM4_ESM.tif]

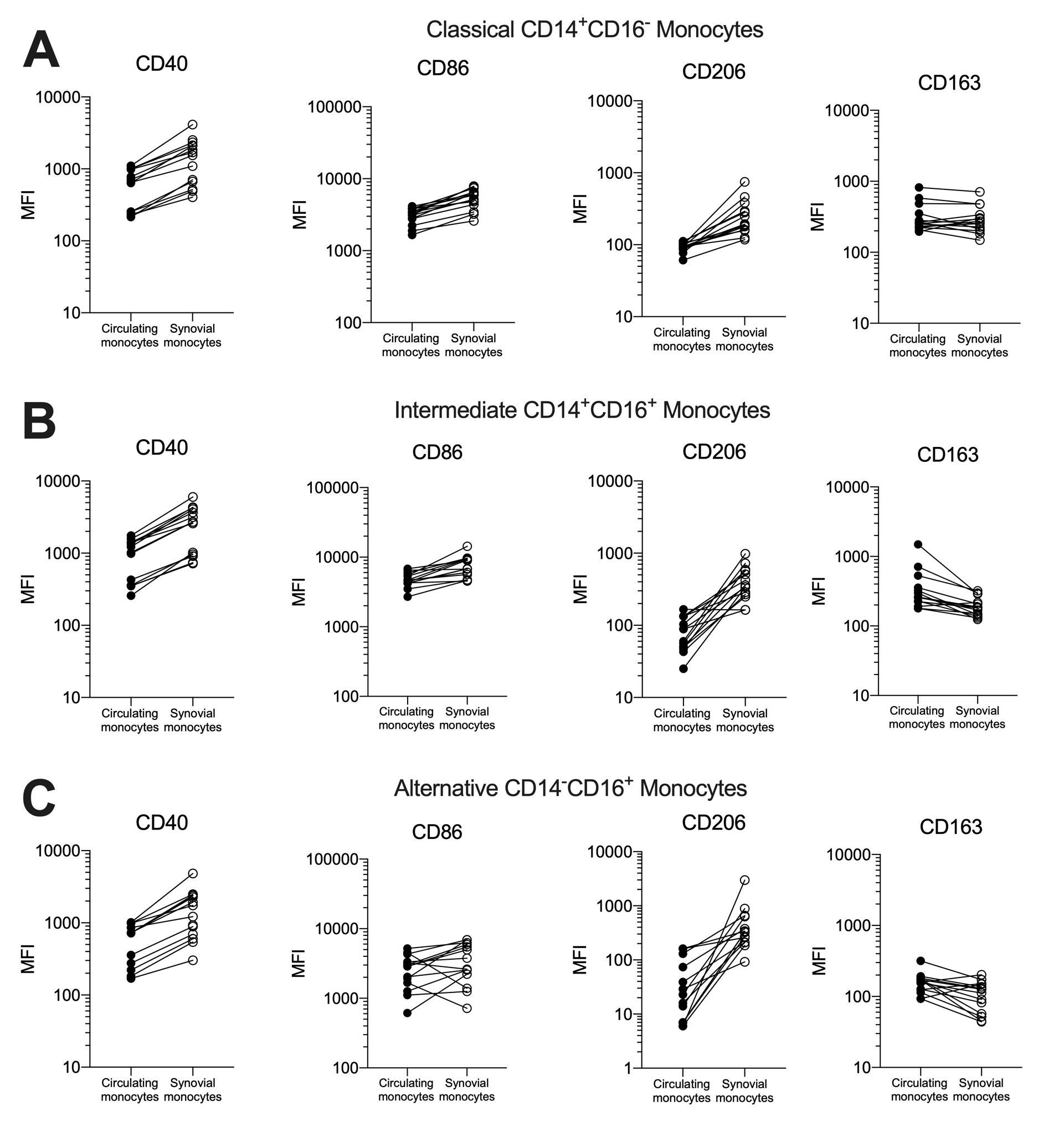

Supplement: Supplementary file 5 — Additional file 5: Supplementary figure 4. Expression of polarization markers across monocyte subsets. Shows the MFI values of each investigated polarization markers in circulating vs synovial populations of (A) CD14+CD16− ‘classical’ monocytes (B) CD14+CD16+ ‘intermediate’ monocytes and (C) C14−CD16+ ‘alternative’ monocytes. [file 13075_2020_2279_MOESM5_ESM.tiff]

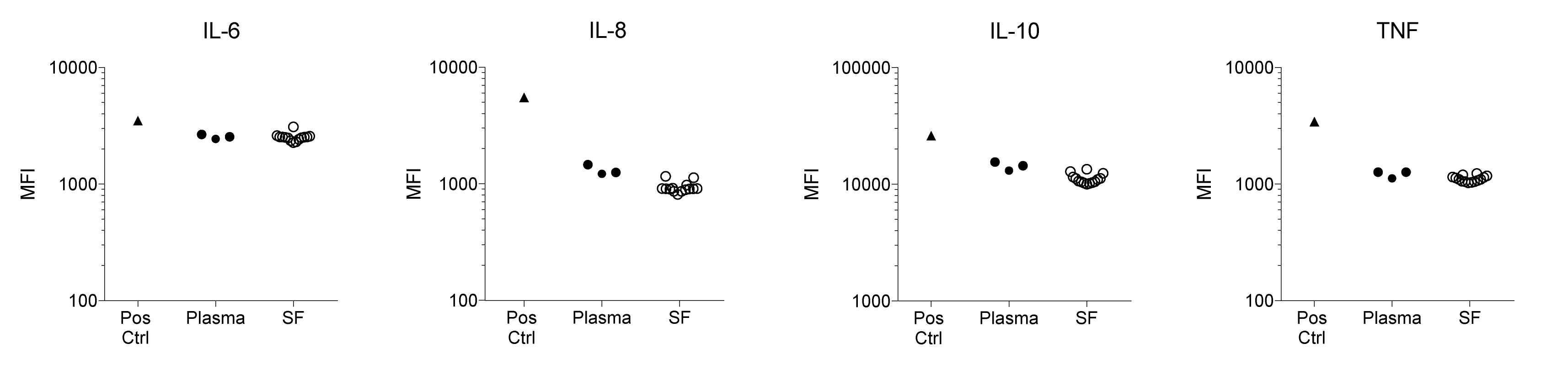

Supplement: Supplementary file 6 — Additional file 6: Supplementary figure 5. Activation of cytokine production in healthy monocytes by synovial fluid. Monocytes from healthy donors were stimulated with 20% synovial fluid or 20% plasma from healthy controls. Synovial fluid did not induce production of IL-6, IL-8, IL-10 or TNF in healthy monocytes as compared to plasma from healthy donors. LPS (1 ng/ml) was used as positive control. SF n = 13, plasma n = 3. [file 13075_2020_2279_MOESM6_ESM.tif]

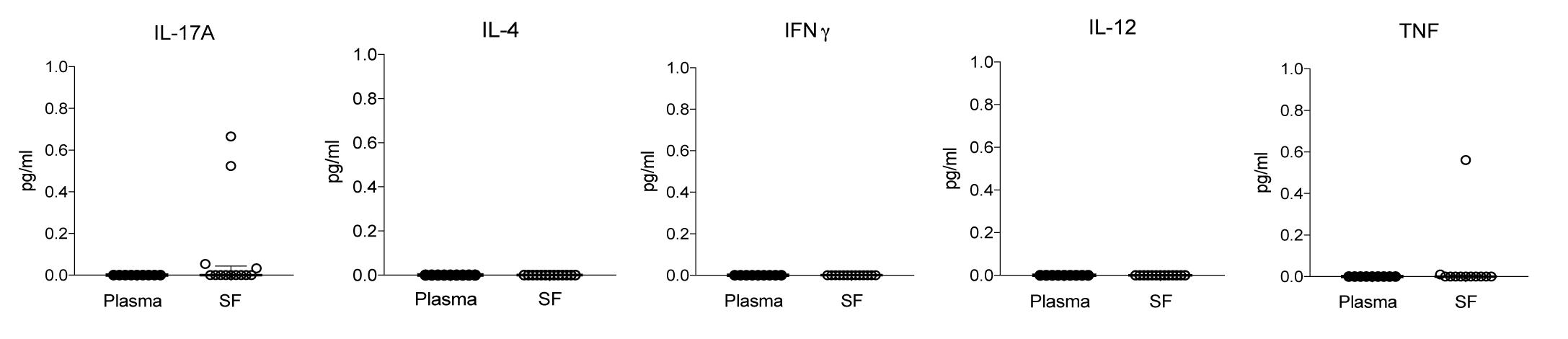

Supplement: Supplementary file 7 — Additional file 7: Supplementary figure 6. Additional cytokine measurements in patients with oligoarticular JIA. Cytokines were measured in synovial fluid and plasma from patients with oligoarticular JIA. The cytokines concentration of IL-17A, IL-4, IFNγ, IL-12 and TNF in plasma were below detection limit in most of the patients. [file 13075_2020_2279_MOESM7_ESM.tif]

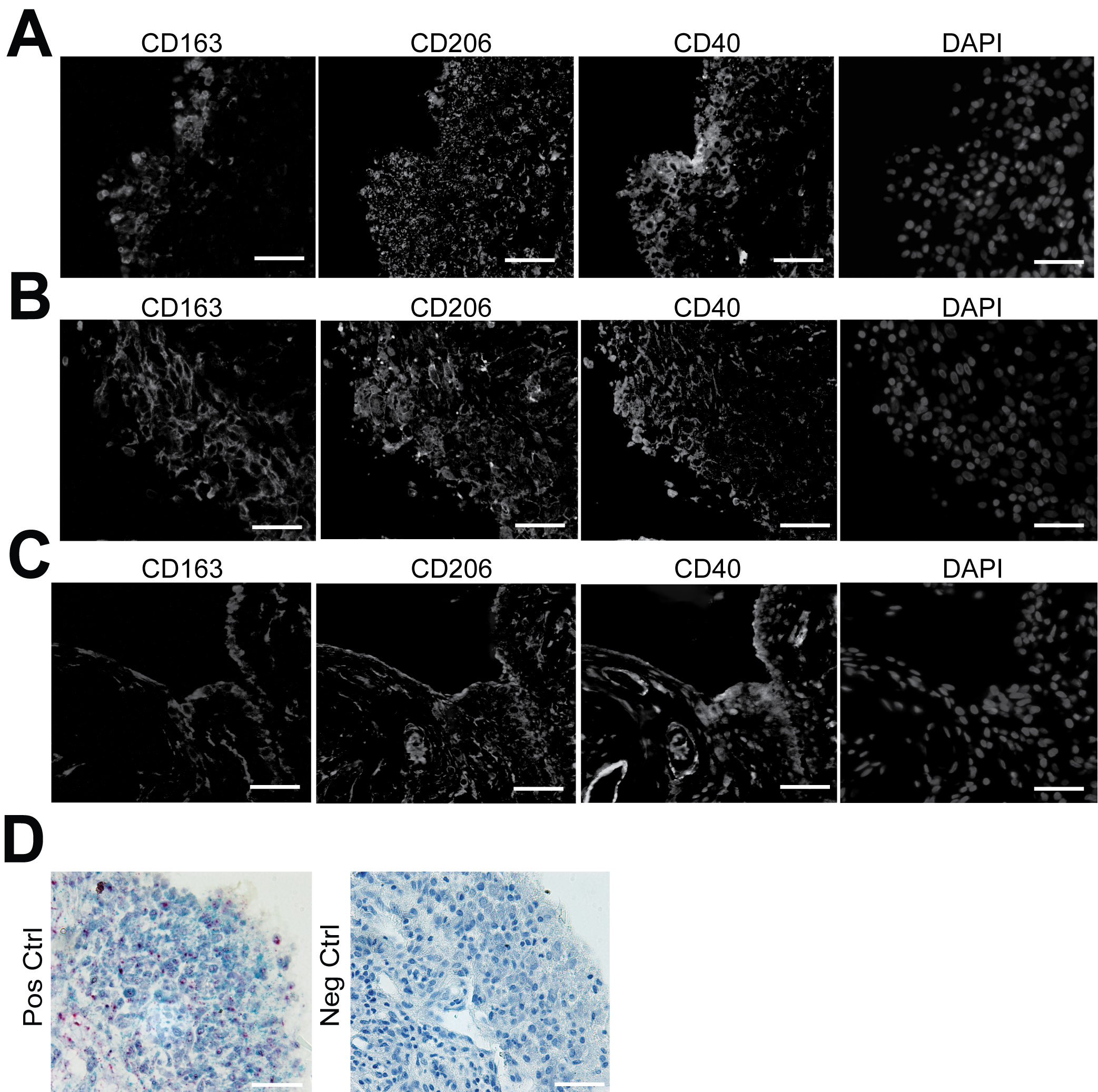

Supplement: Supplementary file 8 — Additional file 8: Supplementary figure 7. Grayscale images of the investigated polarization markers and in situ hybridization controls. (A-C) individual channels in grayscale of CD163, CD206, CD40 and DAPI in the three synovial biopsies analyzed (patient 1, 3 and 8, respectively). (D) Representative images of positive and negative controls of the in situ hybridization experiments. Scale bar: 50 μm. [file 13075_2020_2279_MOESM8_ESM.tif]
